# Ameliorating effect of olive oil on fertility of male rats fed on genetically modified soya bean

**DOI:** 10.3402/fnr.v59.27758

**Published:** 2015-09-18

**Authors:** Thanaa A. F. El-Kholy, Hatim A. Al-Abbadi, Dina Qahwaji, Ahmed K. Al-Ghamdi, Vishal G. Shelat, Hanan M. Sobhy, Mohammad Abu Hilal

**Affiliations:** 1 Clinical Nutrition Department, Faculty of Applied Medical Sciences, King Abdulaziz University Hospital, Jeddah, Saudi Arabia; 2 Surgery Department, King Abdulaziz University Hospital, Faculty of Medicine, Jeddah, Saudi Arabia; 3 Director of Experimental Surgery Unit, King Fahd Medical Research Center, King Abdulaziz University, Jeddah, Saudi Arabia; 4 Department of Medical Laboratory Technology, Faculty of Applied Medical Sciences, King Abdulaziz University, Jeddah, Saudi Arabia; 5 School of Medicine, University of Southampton, UK; 6 Biochemical, Toxicology and Food Deficiency Department, Animal Health Research Institute, Dokki, Gizza

**Keywords:** fertility, genetic modification, olive oil, soya bean

## Abstract

**Background:**

Genetically modified soya bean (GMSB) is a commercialized food. It has been shown to have adverse effects on fertility in animal trials. Extra virgin olive oil (EVOO) has many beneficial effects including anti-oxidant properties. The aim of this study is to elucidate if addition of EVOO ameliorates the adverse effects on reproductive organs of rats fed on GMSB containing diet.

**Methods:**

Forty adult male albino rats (150–180 g) of Sprague Dawley strain were separated into four groups of 10 rats each: Group 1 – control group fed on basal ration, Group 2 – fed on basal ration mixed with EVOO (30%), Group 3 – fed on basal ration mixed with GMSB (15%), and Group 4 – fed on basal ration mixed with GMSB (15%) and EVOO (30%). This feeding regimen was administered for 65 days. Blood samples were collected to analyze serum zinc, vitamin E, and testosterone levels. Histopathological and weight changes in sex organs were evaluated.

**Results:**

GMSB diet reduced weight of testis (0.66±0.06 vs. 1.7±0.06, *p*<0.001), epididymis (0.489±0.03 vs. 0.7±0.03, *p*<0.001), prostate (0.04±0.009 vs. 0.68±0.04, *p*<0.001), and seminal vesicles (0.057±0.01 vs. 0.8±0.04, *p*<0.001). GMSB diet adversely affected sperm count (406±7.1 vs. 610±7.8, *p*<0.001), motility (*p*<0.001), and abnormality (*p*<0.001). GMSB diet also reduced serum zinc (*p*<0.05), vitamin E (*p*<0.05), and testosterone (*p*<0.05) concentrations. EVOO diet had no detrimental effect. Addition of EVOO to GMSB diet increased the serum zinc (*p*<0.05), vitamin E (*p*<0.05), and testosterone (*p*<0.05) levels and also restored the weights of testis (1.35±0.16 vs. 0.66±0.06, *p*<0.01), epididymis (0.614±0.13 vs. 0.489±0.03, *p*<0.001), prostate (0.291±0.09 vs. 0.04±0.009, *p*<0.001), seminal vesicle (0.516±0.18 vs. 0.057±0.01, *p*<0.001) along with sperm count (516±3.1 vs. 406±7.1, *p*<0.01), motility (*p*<0.01), and abnormality (*p*<0.05).

**Conclusion:**

EVOO ameliorates the adverse effects of GMSB on reproductive organs in adult male albino rats. This protective action of EVOO justifies its use against the oxidative damage induced by GMSB in reproductive organs.

Plant foods provide a major source of nutrition for humans. Food shortage is a growing concern. Genetic modification of plants serves four purposes: 1) increase in production, 2) herbicide tolerance, 3) insecticide resistance, and 4) improved nutritional characteristics. The safety and nutritional assessment of genetically modified plant food are not widely reported. Genetic modification of plant food can lead to unintended effects on plant which can have environmental impact, affect animal and human safety and nutritional quality. Genetically modified soya bean (GMSB) has been commercialized in many markets (1). Soya bean contains isoflavones, genistein, and daidzein. GMSB can adversely affect sperm quality and quantity in rats and serum testosterone in men (2–4). Extra virgin olive oil (EVOO) is obtained from the olive (*Olea europaea*), traditional tree crop of the Mediterranean. It is used in cooking, pharmaceutical, and cosmetic skin care products. EVOO is considered healthy because of its oleic acid, palmitic acid, and other fatty acids along with traces of squalence and sterols. EVOO has anti-inflammatory, anti-thrombotic, antihypertensive, anti-oxidant, and vasodilator effects (5). The aim of the present study was to verify the possible protective effect of EVOO against GMSB-induced disruption in reproductive system in male rats.

## Material and methods

### Plant material

Soya bean grits, a granular product of soya bean, and EVOO were purchased from the local market in Cairo, Egypt. GMSB and EVOO were prepared freshly and mixed with basal ration for rats at 15 and 30% concentration, respectively (6). The GMSB used in our experimentation differs from wild-type soya bean by being glyphosate resistant and increasing oleic acid content. Diffuse light absorption spectroscopy was used to confirm the purity of EVOO using the quality parameters as described by Mignani et al. (7).

### Animals

Forty adult male albino rats (150–180 g) of Sprague Dawley strain were obtained from the laboratory animal house of Ophthalmic Research Institute, Giza. Rats were acclimatized to laboratory conditions and fed on a ration containing the essential vitamins and trance elements. Drinking water was supplied *ad-libitum*. The rats received standard laboratory environmental conditions (8).

### Experimental design

This was planned to study the effect of EVOO and GMSB on fertility as well as their effect on relevant serum parameters in rats. Using the random number table of Pocock (9), the rats were randomized into four groups (each of 10) as follows: Group 1, fed on basal ration (control group); Group 2, fed on basal ration mixed with EVOO (30%); Group 3, fed on basal ration mixed with GMSB (15%); and Group 4, fed on basal ration mixed with GMSB (15%) and EVOO (30%). Groups 1 and 2 were assigned when random number generated was 1–5, and Groups 3 and 4 were assigned when random number generated was 6–9. Further again Groups 1 and 3 were separated from Groups 3 and 4 when odd and even numbers were generated, respectively. As length of complete spermatogenic cycle is 48–52 days in rats, the study was terminated after 65 days. After 65 days, the body weight was measured and weight gain/loss was charted as a percentage of the total body weight (10). The person doing testis weight and sperm analysis was blinded to the group allocation. The person doing serum analysis was also blinded to the group allocation.

### Sampling

After 65 days, the blood sample was obtained from orbital plexuses and received into dry clean tubes. Samples were left to clot at room temperature for about 2 h, stored overnight in a refrigerator at 4°C, and centrifuged at 3,000 rpm for 15 min. Serum samples were drawn in dry cleaned capped bottles and kept in a deep freeze. Serum zinc, vitamin E, and testosterone levels were measured as per standard techniques described previously (11–13). The measurement of serum zinc was done by 50/50 nitric/perchloric acid digestion using an inductively coupled plasma atomic emission spectrometer (ICP/AES) (Applied Research Laboratories, Dearborn, MI, USA; model 34,000 simultaneous ICP). The detection limits of the ICP-AES for zinc were 0.009 parts per million (ppm). The ICP-AES internal validation was determined by 15 assays on human plasma pools. The standard deviation and coefficient of variation were 0.05 ppm and 5.9%, respectively. A competitive radioimmunoassay (RIA) which uses the I125 isotope as the competing antigen was used to measure total serum testosterone using a dialysis beta counter. The precision for the quality control samples ranged from 14 to 330 ng/dL with a standard deviation of 1.5–27 and % CV of 8–9.5%. The animals were fasted for 12 h prior to sacrificing. The animals were then sacrificed for studying the histopathology of reproductive organs. Testis, seminal vesicles, prostate glands, and epididymis were dissected and weighted in relation to the body weight. The testis was gently manipulated into the abdomen and was removed by excising the mesenteric attachment to the tunica vaginalis. The vas deferens was then severed at the tail of the epididymis and the entire unit freed by excising the blood vessels. The unit thus obtained consisted of the testis, epididymis, and epididymal fat. Next, epididymal fat was separated. Finally, epididymis was then separated from the testis, and heaviest of the testis was weighted and fixed in 10% formalin-saline and stained with hematoxylin and eosin stain (14). The epididymal sperm count, motility, and abnormalities were performed as described by Bearden and Fluuquary (15). We summarized clinical characteristics and study results using proportions and means. Data were expressed as mean±SD. Differences between the mean values of the study variables were tested by the student's *t*-test. For categorical variables, we used chi-square test to determine differences in proportions between groups. Level of significance was kept at 0.05, and SPSS version 14 statistical software package was used for analysis.

## Results

**Fig. 1:**
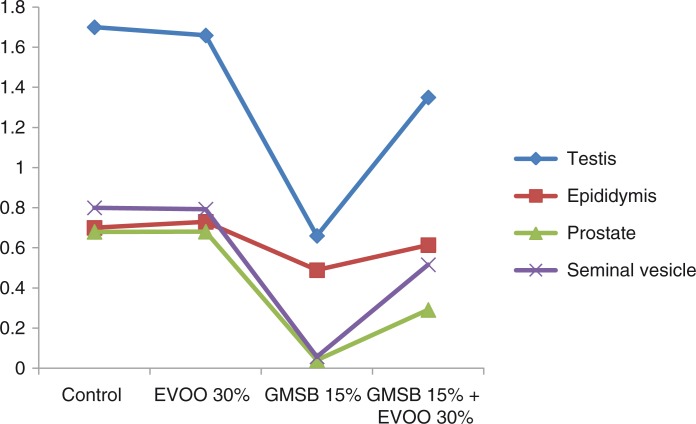
Effect of olive oil and genetically modified soya bean on sex organ weight.

**Fig. 2:**
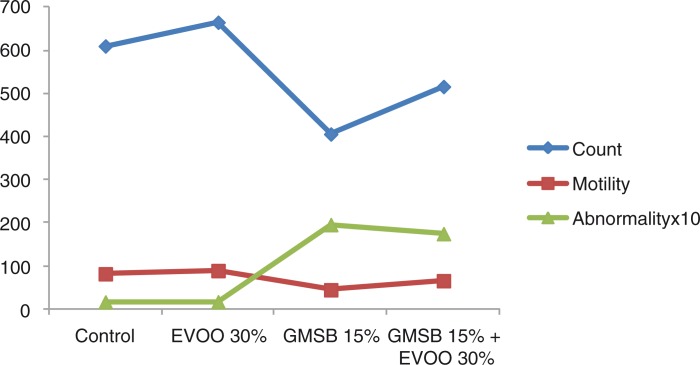
Effect of olive oil and genetically modified soya bean on epididymal sperm characters.

**Fig. 3:**
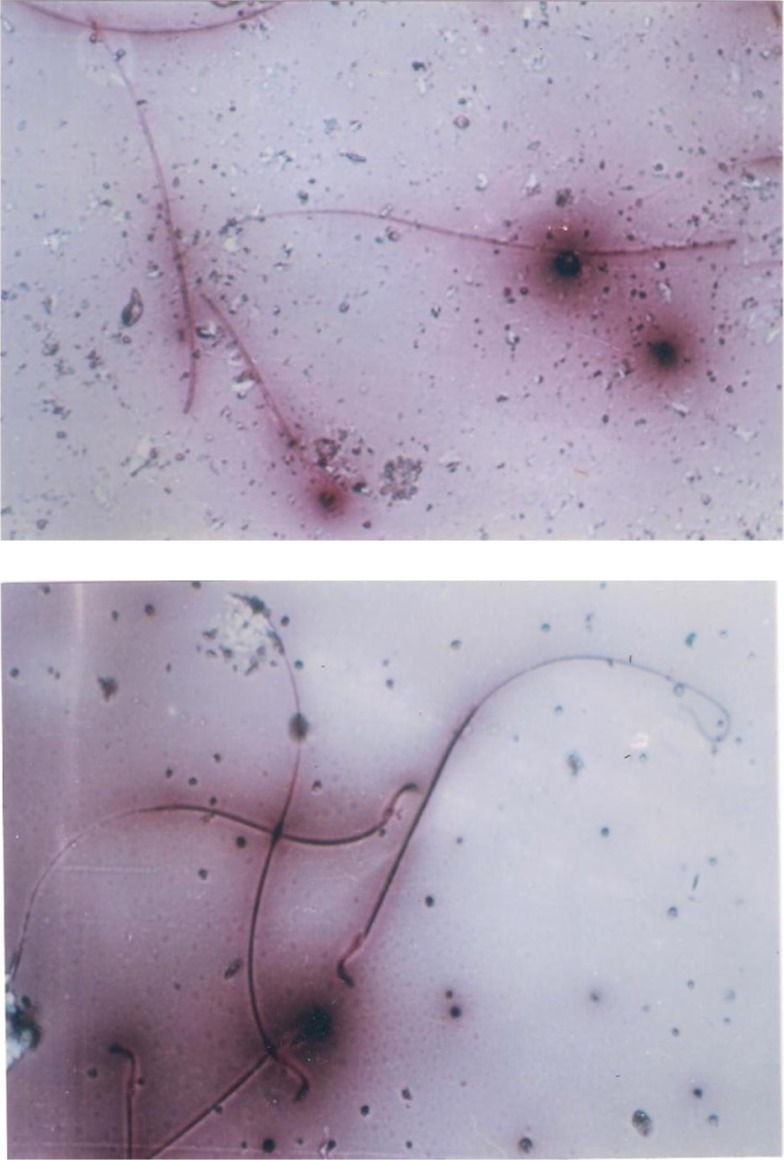
Headless sperm and bent sperm tail after fed on genetically modified soya bean (×100) (eosin-nigrosin stain).

**Fig. 4:**
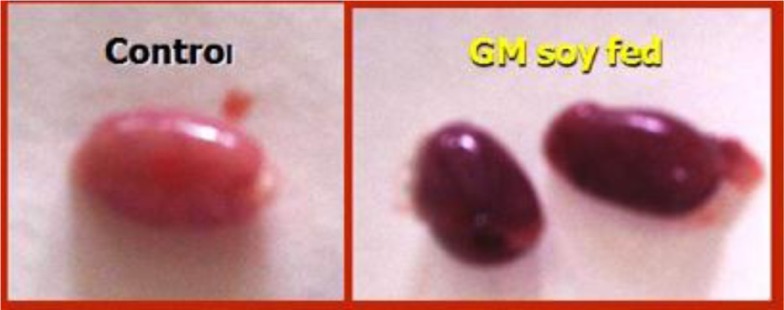
Testicles of rats fed on genetically modified soya bean were smaller and darker in color compared to those fed on normal ration (×100) (eosin-nigrosin stain).

**Fig. 5:**
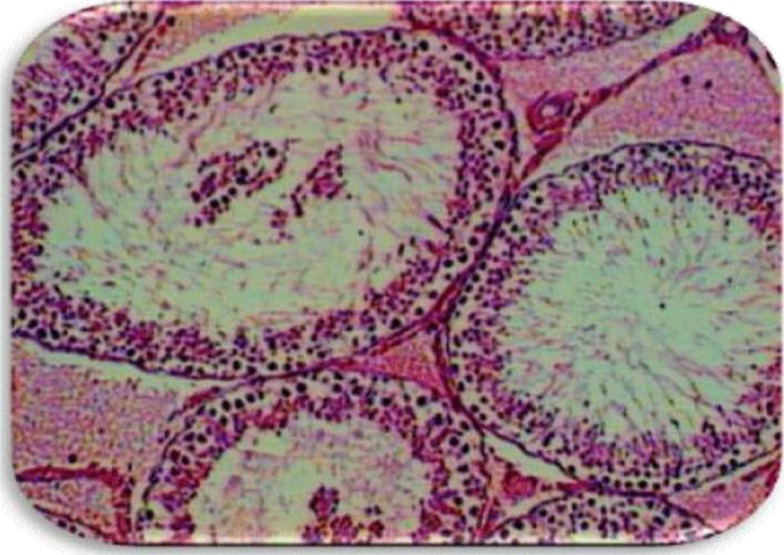
Epididymis of genetically modified soya-bean-fed rats showing immature sperm and degenerated seminal products.

EVOO (30%) fed rats maintained the weight of reproductive organs as compared to control group. GMSB (15%) fed rats manifested weight reduction of testes (*p*<0.001), epididymis (*p*<0.001), prostate glands (*p*<0.001), and seminal vesicles (*p*<0.001) in comparison to all the other groups (Table 1; Fig. 1). Addition of EVOO (30%) to GMSB (15%) diet restored the weights of testis (1.35±0.16 vs. 0.66±0.06, *p*<0.01), epididymis (0.614±0.13 vs. 0.489±0.03, *p*<0.001), prostate (0.291±0.09 vs. 0.04±0.009, *p*<0.001), and seminal vesicle (0.516±0.18 vs. 0.057±0.01, *p*<0.001). GMSB (15%) also decreased sperm count (*p*<0.001) and increased the sperm abnormalities (*p*<0.001) characterized by bent tail and detached head (Table 2; Figs. 2 and 3). Sperm count (516±3.1 vs. 406±7.1, *p*<0.01), sperm motility (66±5.3 vs. 45±3.4, *p*<0.01), and sperm cell abnormality (17.5±0.21 vs. 19.6±0.09, *p*<0.05) were also restored in Group 4. GMSB (15%) fed rats had a significant decrease in body weight while Group 4 rats showed an improvement in the weight gain (Table 3). Figure 4 demonstrates the smaller and darker testis of rats fed on GMSB (15%) diet compared to rats fed on normal diet. Histopathological examination of testes showed degeneration of Leydig cells as well as the spermatogenic cells along with interstitial edema and degeneration of sperms in group fed on GMSB (Fig. 5).

**Table 1:** Effect of olive oil and genetically modified soya bean on sexual organ weight

	Weight of sex organ (g) per 100 g body weight			
	
Group *n*=10 in each group	Testis	Epididymis	Prostate	Seminal vesicle
Control – Group 1	1.7±0.06	0.7±0.03	0.68±0.04	0.8±0.04
EVOO 30% – Group 2	1.659±0.18	0.73±0.09	0.681±0.13	0.793±0.19
GMSB 15% – Group 3	0.66***±0.06	0.489***±0.03	0.04***±0.009	0.057***±0.01
EVOO 30%+GMSB 15% – Group 4	1.35**±0.16	0.614***±0.13	0.291***±0.09	0.516***±0.18

Values are mean±SD.

** *p*<0.01 and

*** *p*<0.001.

**Table 2:** Effect of olive oil and genetically modified soya bean on epididymal sperm characters

Group *n*=10 in each group	Sperm cell count (10^6^/mm^3^)	Sperm motility %	Sperm abnormality %
Control – Group 1	610±7.8	82±5.1	1.66±0.06
EVOO 30% – Group 2	665***±3.4	89.5±4.7	1.7±0.06
GMSB 15% – Group 3	406***±7.1	45***±3.4	19.6***±0.0.9
EVOO 30%+GMSB 15% – Group 4	516**±3.1	66***±5.3	17.5**±0.21

Values are mean±SD

** *p*<0.01 and

*** *p*<0.001.

**Table 3:** Food intake (g) and body weight gain (g) of rats

Group *n*=10 in each group	Food intake (g)	Body weight gain (g)
Control – Group 1	3,500±31.56	50.0±1.06
EVOO 30% – Group 2	4,500±92.61	64.0±0.61
GMSB 15% – Group 3	5,300±120.03	42.4±3.7
EVOO 30% + GMSB 15% – Group 4	5,200±110.08	49.5*±2.2

Values are mean±SD.

* *p*<0.05.

**Fig. 6:**
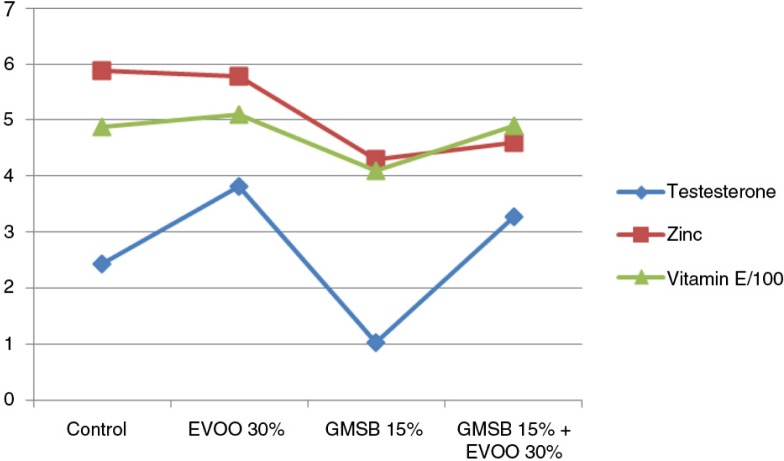
Effect of olive oil and genetically modified soya bean on testosterone, zinc, and vitamin E.

EVOO (30%) fed rats demonstrated an increase in vitamin E (509.76±5.809 vs. 486.87±7.149, *p*<0.05) and testosterone levels (3.82±0.063 vs. 2.44±0.002, *p*<0.05) compared to control group. GMSB (15%) fed rats showed a reduction in zinc (*p*<0.05), vitamin E (*p*<0.05), and testosterone levels (*p*<0.05) when compared with all the other groups (Table 4). Addition of EVOO to the GMSB diet increased the levels of vitamin E (490.60±4.07 vs. 486.87±7.149) and testosterone (3.27±0.172 vs. 2.44±0.002) more than the control group while zinc levels remained below the control group (4.60±0.126 vs. 5.89±0.056) but with a significant increase in comparison to the GMSB-fed rats (4.60±0.126 vs. 4.30±0.21, *p*<0.05) (Fig. 6).

**Table 4:** Effect of olive oil and genetically modified soya bean on serum zinc, vitamin E, and testosterone

Group *n*=10 in each group	Zinc µg/dL	Vitamin E µg/ml	Testosterone ng/ml
Control – Group 1	5.89±0.056	486.87±7.149	2.44±0.002
EVOO 30% – Group 2	5.78±0.274	509.76*±5.809	3.82*±0.063
GMSB 15% – Group 3	4.30*±0.21	409.24*±7.396	1.03*±0.091
EVOO 30% + GMSB 15% – Group 4	4.60*±0.126	490.60*±4.07	3.27*±0.172

Values are mean±SD.

* *p*<0.05.

## Discussion

Consumption of GMSB diet adversely affects the fertility in adult male albino rats. Our results suggest that addition of EVOO (30%) to the GMSB (15%) diet ameliorates the adverse effects on fertility characteristics due to GMSB. These findings do suggest that the addition of EVOO to all the food containing GMSB may be considered.

GMSB has been shown to suppress Leydig cell activity and decrease testosterone production (16, 17). Phytoestrogens within the GMSB may also affect the release of testosterone (4). It has been shown that GMSB reduces the levels of zinc and vitamin E (18–20). Our study shows that zinc, vitamin E, and testosterone levels are low in the adult male albino rats fed on diet containing GMSB at 15% concentration. EVOO is widely known for its anti-inflammatory and anti-oxidant properties. EVOO is also used in various products varying from oral consumption to skin care cosmetics. We have demonstrated that the addition of EVOO to GMSB diet ameliorates the adverse effects of GMSB on serum concentrations of zinc, vitamin E, and testosterone.

GMSB reduces the sexual organ weight, sperm count, sperm quality, and sperm motility. Testicle of mice fed on GMSB showed reduced sperm development and increase in the incidence of neoplastic lesions (21, 22). In our study, histopathological examination of testes showed degeneration of Leydig cells, spermatogenic cells along with interstitial edema in group fed on GMSB. The significant drop in the body weight and accessory sex organs may be due to the presence of anti-nutrient in the raw soya bean being used, which may cause mal-absorption of amino acids present in soya protein. The presence of anti-nutrient exposes the reproductive system of animals to oxidative stress, which has been liked to almost half of the diagnosed cases of oligospermia in humans (23). As GMSB is a standard food addition for commercial purposes, these findings have far-reaching implications in reducing the fertility of population at large. It is reasonable to find an alternative means that does not affect fertility. It is shown that EVOO increased the testicular and accessory glands weight and sperm cell count (24–26). In our study, the addition of EVOO (30%) to basal ration did not lead to increase in sex organ weight, but increased the sperm count and motility compared to control group. There is no literature to note the effect of addition of EVOO to GMSB diet. Addition of EVOO to rats fed on GMSB diet increased the sexual organ weight, sperm count, sperm quality, and sperm motility. This suggests that EVOO restores the adverse effects of GMSB diet. Non-vitamin anti-oxidant, polyphenols reduced the negative effect of oxidative stress and free radicals in diabetic patients (27). Several mechanisms have been proposed to explain the anti-oxidant capacity of olive oil. One possible mechanism is the increase of tissue sensitivity to the lipid anti-oxidants of vitamin E (28). The diet rich in olive oil reduces tissue oxidative stress. It was showed that the oleic acid, the major component of olive oil has an anti-oxidant effect (29). The polyphenols in olive oil (oleuropein, tyrosol, hydroxytyrosol, and caffeic acid) inhibit the formation of oxygen reactive species in the cells (30). Oleuropein, a phenolic anti-oxidant, can reduce the oxidative stress that results from doxorubicin (31).

It can be concluded that GMSB induces harmful effects in the male reproductive system and relevant biochemical parameters. Adding EVOO to the diet of albino rats is effective in ameliorating these harmful effects which may protect or restore fertility.
